# CD8^+^ DC, but Not CD8^−^DC, Isolated from BCG-Infected Mice Reduces Pathological Reactions Induced by Mycobacterial Challenge Infection

**DOI:** 10.1371/journal.pone.0009281

**Published:** 2010-02-18

**Authors:** Xiaoling Gao, Shuhe Wang, Yijun Fan, Hong Bai, Jie Yang, Xi Yang

**Affiliations:** 1 Laboratory for Infection and Immunity, Department of Medical Microbiology, Faculty of Medicine, University of Manitoba, Winnipeg, Manitoba, Canada; 2 Department of Immunology, Tianjin Medical University, Tianjin, People's Republic of China; University of Delhi, India

## Abstract

**Background:**

Tuberculosis is a mycobacterial infection causing worldwide public health problems but the available vaccine is far from ideal. Type-1 T cell immunity has been shown to be critical for host defence against tuberculosis infection, but the role of dendritic cell (DC) subsets in pathogenesis of mycobacterial infection remains unclear.

**Methodology/Principal Findings:**

We examined the effectiveness of dendritic cell (DC) subsets in BCG-infected mice in generating immune responses beneficial for pathogen clearance and reduction of pathological reactions in the tissues following challenge infection. Our data showed that only the adoptive transfer of the subset of CD8α^+^ DC isolated from infected mice (iCD8^+^ DC) generated significant protection, demonstrated by less mycobacterial growth and pathological changes in the lung and liver tissues in iCD8^+^ DC recipients than sham-treated control mice. The adoptive transfer of the CD8α^−^DC from the infected mice (iCD8^−^ DC) not only failed to reduce bacterial growth, but enhanced inflammation characterized by diffuse heavy cellular infiltration. Notably, iCD8^−^ DC produced significantly higher levels of IL-10 than iCD8^+^ DC and promoted more Th2 cytokine responses in in vitro DC-T cell co-culture and in vivo adoptive transfer experiments.

**Conclusions/Significance:**

The data indicate that in vivo BCG-primed CD8^+^ DC is the dominant DC subset in inducing protective immunity especially for reducing pathological reactions in infected tissues. The finding has implications for the rational improvement of the prophylactic and therapeutic approaches for controlling tuberculosis infection and related diseases.

## Introduction

Tuberculosis is a type of mycobacterial infection that causes serious public health problems worldwide. The World Health Organization (WHO) estimates that about 2 billion people, meaning one third of the world's population, are infected with *Mycobacterium tuberculosis* (Mtb) [1], [2]. More importantly, the incidence of tuberculosis continues to rise due to the spread of HIV infection and the emergence of multi-drug-resistant Mtb strains [3], [4]. Mtb infection is the leading cause of death in AID patients. BCG (Bacille Calmette -Guérin) is the only registered human vaccine for prevention of Mtb infection and diseases. Although its protective effect has been well established, its efficacy is quite variable and far from ideal [5]. A better understanding of the mechanism by which BCG immunization protects host from challenge infection may be not only useful for the possible improvement of the efficacy of this particular vaccine but also have implications in the rational development of new vaccines for tuberculosis.

Although the mechanism remains unclear, the host protection against Mtb infection has been found to be largely dependent on T cell mediated immunity especially type-1 T cell responses. The influence of type-2 T cells and humoral immune responses to host defence is limited and even detrimental in certain circumstances. Human studies have shown that Th1 response is essential for the host protection from mycobacterial infections [6]. The human subjects deficient in receptors for IFN-γ and IL-12 are profoundly susceptible to mycobacterial infections [7], [8]. Experimental studies using animal models also confirmed the critical importance of Th1 responses in protective immunity and the control of pathological reactions (reviewed in [8], [9].

T cell response is a downstream event in the immune system, following the capture, process and presentation of antigenic peptides by antigen-presenting cells (APC). Along with macrophages and others, dendritic cells (DCs) are a type of the front line cells that encounter *Mycobacteria* in the infection sites. DCs are the key APCs in the activation of primary CD4 T cells and in the polarization of Th1/Th2 subsets. It has been shown that Mtb-infected DCs, but not macrophages, can drive naïve CD4^+^ T cells polarize to Th1 pathway [10]. The DCs in the lung play a central role in initiating the immune response to tuberculosis [11]. DCs can influence the polarization of naive T cell to different types of effector T cells [12], [13]. Since DC is a heterogeneous population, different DC subtypes may contribute differently on the activation and polarization of mycobacterial antigen-specific T cells. Indeed, phenotypic and functional alterations in DCs have been reported in individuals with mycobacterial infection[14]. In human, CD16^−^mDC and CD16^+^mDC subsets have been found to be different in preferential activation of T memory cells, showing that CD16^+^mDC elicits stronger IFNγ response than CD16^−^mDC[15]. In mice, splenic CD8α^+^ and CD8α^−^ DC subsets have been extensively studied and some functional differences between the two subsets have been reported in several model systems including infections, although some studies showed overlapping functions of the two subsets [16]–[20]. In particular, we have reported that CD8α^+^ DC isolated from *Chlamydia muridarum* infected mice are more potent in inducing protection against challenge infection with the same Chlamydia species than CD8^−^ DC[21]. Little is known about the functional involvement of DC subsets in BCG induced protective immunity against mycobacterial infection.

In the present study, we examined the relative effectiveness of splenic CD8α^+^(CD8^+^) and CD8α^−^ (CD8)DC subsets in BCG-infected mice in generating type-1 T cell responses and protection against challenge infection with an intention to know more on the mechanism by which a host defenses against Mtb infection. The data showed that the distinct DC subsets primed by in vivo BCG immunization are significantly different in their potency to induce protective immunity against challenge mycobacterial infection. Specifically, we found that CD8α^+^ DC is much more potent than CD8α^−^ DC for inducing type-1 T cell responses in both in vitro and in vivo conditions. This difference is unlikely due to the potential difference of the subsets in uptaking mycobacterial organisms because quantitative RT-PCR test showed similar levels of bacterial messages in the isolated DC subsets. Rather, it is more likely due to the functional difference of the DC subsets in modulating T cell responses.

## Materials and Methods

### Mice and Culture Medium

Female C57BL/6 mice were bred at the University of Manitoba breeding facility and hosted under specific-pathogen-free conditions. Animals were used in accordance with the guidelines issued by the Canadian Council on Animal Care. The animal experimental protocol was approved by the ethical committee of The University of Manitoba.

Complete RPMI 1640 medium supplemented with 10% heat–inactivated fetal bovine serum, 1%L-glutamine, 25 µg/ml gentamycin and 5×10^−5^M 2-mercaptoethanol was used for cell culture.

### Organism

The original BCG vaccine was produced by Aventid Pasteur Limited (Toronto, Ontario, Canada). For expansion of the vaccine, BCG was grown in the Middlebrook's 7H9 broth (Difco Laboratories Inc., Detroit, MI) containing 0.2%(v/v) glycerol and 0.05%(v/v)Tween-80 and supplied with 10%(v/v) Middlebrook ADC enrichment (Difco) for 21days. The number of BCG bacilli, counted as colony-forming units (CFU), was measured by plating diluted culture on plates of Middlebrook 7H11 agar (Difco) containing 0.5%(v/v)glycerol and supplied with Middlebrook OADC enrichment(Difco). The BCG stock was stored at −80°C until use. For inactivation of BCG, live BCG was put at 65°C for 1 hour, which led to complete killing of BCG confirmed by viability testing.

### Mice Immunization

Mice were injected intravenously with 5×10^5^ CFU BCG in 200 µL sterile protein-free PBS and sacrificed at day 21 after infection. Spleens were aseptically isolated and treated as following: Briefly, the spleens were digested in 1.5 mg/ml collagenase D at 37°C for 30 min and EDTA was applied at the last 5 min to disrupt DC-T complexes. The cell suspensions was then pipetted up and down several times and filtered.

### DC Subset Isolation

For total DC (CD11c^+^) isolation, the splenocytes after RBC lyses with ACK lyses buffer(150 mM NH_4_Cl, 10 mM KHCO_3_, 0.1 mM EDTA) were incubated with the MACS (Miltenyi Biotec, Auburn, CA) CD11c beads for 15 min at 4°C. The cells were washed and passed through the LS columns for selection of CD11c^+^ cells. For isolation of DC subset (CD11c^+^CD8^+^DC and D11c^+^CD8^−^DC), MACS CD8^+^DC positive selection kit was used according to the manufacturer's instructions. Briefly, spleen cells were incubated with cocktail of biotin-conjugated antibodies against antibodies (CD90, CD45R, CD49b), followed by incubation with anti-biotin microbeads to deplete of T, B and NK cells. The CD8^+^DC subset was isolated from the T,B and NK cell depleted preparation by incubating it with CD8^+^ cell selecting beads for 30 min on ice and passing through LD columns. The CD8^−^DC subset was further isolated from this preparation using CD11c column. The purity of the total DC and DC subset was >95% based on flow cytometry analyses.

### Flow Cytometric Analysis

For analysing total DC, purified CD11c^+^ DCs (2×10^6^ the cells) were pre-incubated with anti-mouse CD16/32 mAb for 15 min to block FcR binding before staining with specific antibodies. Cells were then incubated with specific mAbs at 4°C for 30 min in dark. After washing with a staining buffer [Dulbecco's PBS (Sigma-Aldrich) without Ca^2+^ and Mg^2+^ containing 2% heat-inactivated FCS and 0.05% NaN_3_], the cells were fixed and analysed using FACS Calibur flow cytometer (BD Biosciences) and the data were analyzed using Cell Quest™ software (Becton Dickinson). To analyzed surface marker expression on DC subsets, three color-staining was performed. CD11c-allophycocyanin (Hamster IgG) and anti CD8-PE-Cy7 (Rat IgG2a κ) were used for gating DC subsets and FITC conjugated anti-CD80 (Hamster IgG), CD86 (Rat IgG2b κ), CD40 (Rat IgG2a κ) and MHC-II (Rat IgG2b) were used for the specific markers. The florescence-conjugated appropriate isotype controls antibodies were used as control.

Intracellular cytokines were analyzed as we previously described[22]. Briefly, freshly isolated draining lymph node cells (2×10^6^ cells) were stimulated with 50 ng/ml phorbol 12-myristate 13-acetate (PMA, Sigma Chemical Co., USA) and 1 µg/ml ionomycin (Sigma) for 6 hours. 20 µg/ml brefeldin A (Sigma) was added in the last 3 hours in order to accumulate cytokines intracellularly. After washing with a staining buffer, cells were incubated with anti-mouse CD16/32 (Fc block, e-Bioscience) for 30 min on ice to block the FcR non-specific binding and subsequently stained for surface markers with PE-anti-CD4, FITC-anti-CD3ε, PerCy-anti-CD8 or isotype controls for 30 min on ice. The cells were fixed and permeabilized with Cytofix/Cytoperm™ buffer (BD BioScience) for 20 minutes at 4°C and incubated with allophycocyanin anti-IFN-γ or corresponding isotype controls (eBioscience) for 20 minutes on ice. The raw sample data were collected using a FACS Calibur flow cytometer (BD Biosciences) and the data were analyzed using FlowJo (BD Biosciences).

### Adoptive Transfer of DC Subsets and Challenge Infection

Two models were used. For model 1, intravenous route was used. Purified CD8^+^ DCs and CD8^−^ DCs from BCG infected mice were injected intravenously (i.v.) to syngeneic recipient mice at the amount of 5×10^5^ cells in 200 µl sterile protein-free PBS. The mice only received the PBS (sham-treatment) or DC subsets from naive mice were used as control groups. At day 7 after adoptive transfer, the mice were challenged with 5×10^5^ CFU BCG i.v. and were sacrificed at day 21 after challenge. The lungs and livers were homogenized in 10 ml PBS and plated in serial dilutions onto the Middlebrook 7H11 agar with Middlebrook ADC enrichment. The culture was allowed to proceed for 21 days at 37°C in an atmosphere of 9% CO_2_ and the number of CFUs was counted. For model 2, the DC subsets were adoptively transferred to recipient mice by intranasal (i.n.) route at the amount of 2×10^5^ cells in 40 µl sterile protein-free PBS. Two hours later, the recipient mice were challenged with 2×10^5^ CFU BCG i.n. and killed at day 21 after challenge infection. The BCG CFU in the lung homogenates were measured as above.

### Histopathological Analysis

Lung and liver tissues of the mice with challenge infection were routinely fixed in 10% buffered formalin, embedded in paraffin, sectioned by a microtome, stained by hematoxylin and eosin (H&E) and examined under a light microscope. Infiltrating inflammatory cells were identified based on cellular morphology and characteristics.

### Cytokine Response Analysis

For analysing cytokine production by cultured spleen cells, single spleen cells (7.5×10^6^ cells) were cultured in 1 ml complete medium for 72 h in the presence of heat-killed (HK)-BCG (7.5×10^5^ CFU). Cytokines in the supernatants were measured using ELISA. For testing cytokine levels in local tissues, the lung and liver tissues were homogenized in the 10 ml cold PBS. The supernatant were collected after centrifugation and the cytokine were measured by the ELISA.

To test the spontaneous cytokines production by the freshly isolated DC subsets, the DC subsets cells were cultured with complete medium using 96-well culture plates at 5×10^5^ cells/well for 72 hours. The level of IL-12p70 and IL-10 in the culture supernatants were measured by ELISA.

### DC Subsets–T Cell Co-Culture

To directly assess the T cell modulating abilities of DC subsets, purified DC subsets were cultured with CD4 T cells isolated from BCG-infected mice as we previously described[22]. CD4 T cells were isolated from the spleen using the CD4 T cell isolation column from MACS. The purity of CD4 T cells were >95%. DC subsets (1×10^5^ cells/well) and CD4 T cells (1×10^6^ cells/well) were co-cultured in 96-well plates in the presence of HK-BCG (5×10^4^ CFU/well) in 200 µL complete RPMI medium for 72 hours and the concentration of IFN-γ and IL-4 in the supernatants were measured by ELISA.

### Quantitation of BCG Gene Expression in DCs by Real-Time RT-PCR

To analyze the BCG gene expression in the isolated DC subsets from BCG infected mice, real-time RT-PCR were performed. cDNA synthesis is performed using total mRNA primed with random primer. The thermal profile for RT was incubated at 25°C for 5 min, 50°C for 1 hour. Inactivation of the reaction was done by heating at 70°C for 15 min. The real-time RT-PCR was carried out on the MiniOpticon™ System using an iG™ SYBR Green Supermix (BioRad Laboratories, Hercules, California, USA).The PCR was performed in a 48-well plate in a reaction volume of 25 µl containing 12.5 µl 2x iG™ SYBR Green Supermix Mix, 200 nM each of forward and reverse primer, 0.5 µl template cDNA, added nuclease-free water to a final volume of 25 µl. The thermal profile for PCR was 95°C for 10 min, followed by 40 cycles of 95°C for 30 s and 58°C for 30 s, and 72°C for 60 s. Fluorescent signals were read and the data were collected at each annealing temperature. For each sample, the amplification plot and the corresponding dissociation curve were examined. The melting curve analysis, determining the specificity of the reaction, was carried out immediately after the final PCR cycle by measuring the changes in fluorescence during slowly heating the amplicon/probe heteroduplex. The threshold cycle (*Ct*) used in the real-time PCR quantification was defined as the PCR cycle number that a noticeable increase in reporter fluorescence above a baseline signal. The efficiency of target gene and GAPDH is similar and a comparative *Ct* method was used for calculations[23]. GAPDH was used as an endogenous control gene for normalization of the amount of RNA added to the reactions. Δ*Ct* = *Ct*(BCG) - *Ct*(GAPDH), which means the difference between the threshold cycle of BCG and the threshold cycle of the corresponding GAPDH in the same sample. For each experiment, a negative control of nuclease-free water and a positive control (with known *Ct* value) were run in triplicate. The specific primers used in quantitative real-time PCR were: BCG IS6110 (123 bp): 5′CCTGCGAGCGTAGGCGTCGG; 3′CTCGTCCAGCGCCGCTTCGG. GAPDH (191 bp): 5′AACGACCCCTTCATTGAC, 3′CACGACTCATACAGCACCT.

### Statistical Analysis

One-way ANOVA (One-way analysis of variance) and further Newman-Keulse test were used to determined statistic significance among groups. CFU of BCG was converted to logarithmic values and analyzed using the ANOVA test. The value of *P*<0.05 was considered significant.

## Results

### BCG Immunization Induces Preferential Expansion of CD8^+^ DC Subset Which Exhibits Differential Expression of Surface Markers and the Production of Cytokines Compared to CD8^−^ DC Subset

**Figure 1:**
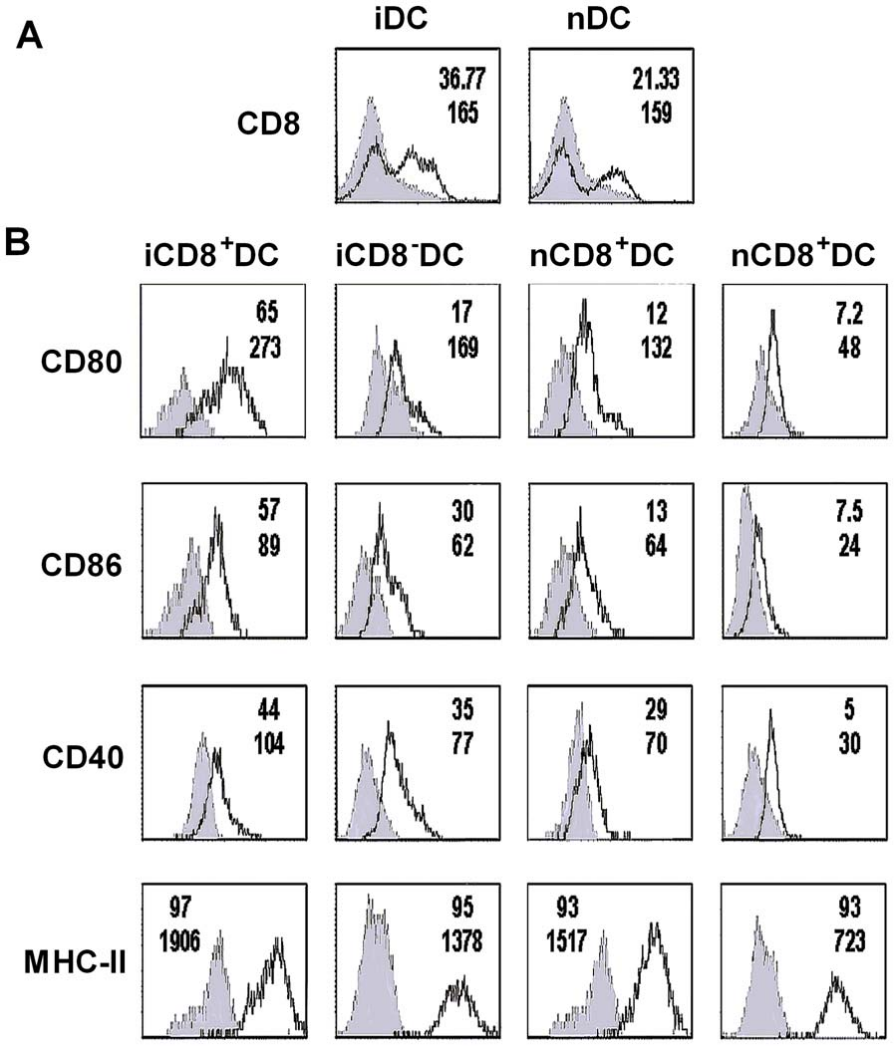
BCG immunization induced the expansion of CD8^+^ DC which expressed higher levels of co-stimulatory molecules compared with CD8^-^ DC. Mice (C57BL/6, n = 4/group) were infected i.v. with 5×10^5^ CFU of BCG and sacrificed at 21 days after immunization. Total DCs from infected and naïve mice were purified using the MACS CD11c^+^ isolation column. Purified DCs were co-stained with APC-conjugated anti-CD11c, PE-Cy7-conjugated anti-CD8 and FITC-conjugated Ab specific for one of the surface markers (CD80, CD86, CD40 or MHCII). The surface marker expression (solid lines) or matched Ab isotype control (shaded histogram) are shown respectively. All histogram were based on 10,000 cells satisfying a gate set of forward vs side scatter light histogram. A, purified DCs were gated on CD11c positive cells showing CD8^+^ DC population in infected (iDC) and naïve (nDC) mice. B, purified DC were gated on either CD11c^+^ CD8^+^ DC (CD8^+^DC) or CD11c^+^CD8^−^ DC (CD8^−^ DC) and the surface molecules on the DC subsets were shown. The percentages of positive cells and mean fluorescence intensity (MFI) for the molecules were shown at the top and bottom lines respectively at the right upper corner of each histogram.

To analyze the effect of BCG immunization on DC subset, we measured CD8 molecule expression on the total DC (CD11c^+^ cells) isolated from the spleens of BCG infected and naive C57BL/6 mice. As shown in Figure 1A, DCs isolated from BCG infected mice showed a higher percentage of CD8^+^ subpopulation than the DCs from naïve mice (37% vs 21%), suggesting a preferential expansion of CD8^+^ DC following BCG immunization. Since the function of DC in modulating immune responses is largely dependent on their expression of co-stimulatory molecules and the production of cytokines, we further analyzed the surface markers on the DC subsets by three-color staining (CD11c, CD8 and a particular surface marker). As shown in Figure 1B, in comparison with the CD8^−^ DC isolated from BCG infected mice iCD8^−^ DC, the CD8^+^ DC isolated from the mice with the same infection iCD8^+^ DC expressed higher CD80 (65.66% vs 17.43%), CD86 (57.43% vs 30%) and CD40 (44% vs 35%) molecules. Similar differences were observed in comparison of the density (mean fluorescence intensity, MFI) of these molecules expressed on the surface of these cells. Although the iCD8^+^ DC and iCD8^−^ DC subsets showed similar MHC-II in percentage (97% vs 95%), the MFI of MHC-II was much higher in iCD8^+^ DC. Similar pattern of differences in expression levels of surface markers were found in naïve DC subsets, but the absolute levels in the naïve mice were lower than those of infected mice, suggesting a significant impact of immunization on both DC subsets.

**Figure 2:**
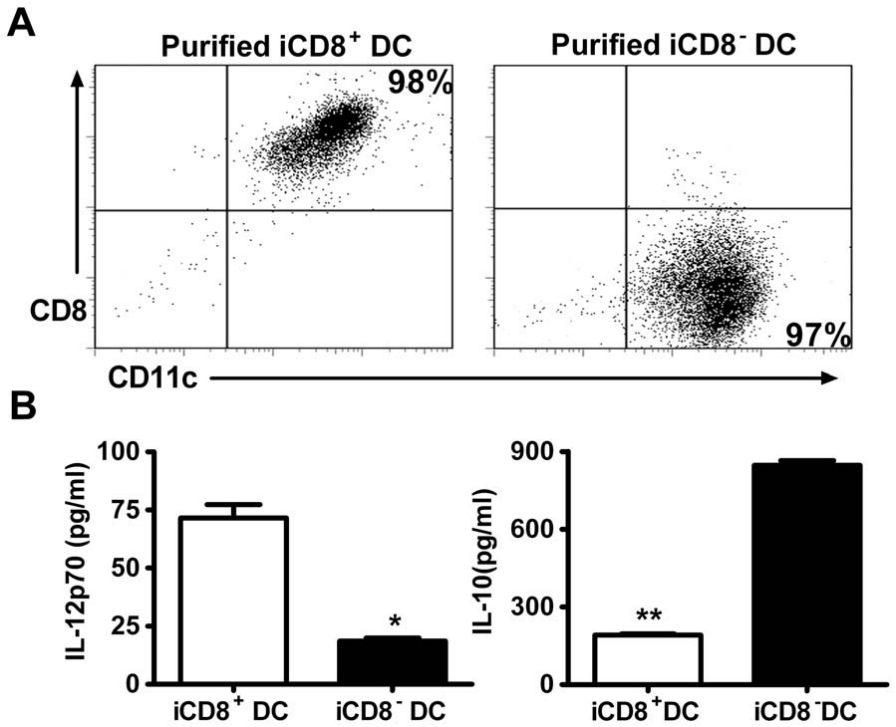
Different levels of cytokine production by DC subsets. DC subsets were isolated from spleens of BCG infected (i.v.) mice at day 21 post-immunization as described in the Materials and Methods. A, purified DC subsets were analyzed by flow cytometry for purity. The 5×10^5^ sorted cells were co-stained with APC-conjugated anti-CD11c and PE-conjugated anti-CD8. The purities of the sorted iCD8^+^ DC (left) and iCD8^−^DC (right) are indicated at the right upper corner and right lower corner respectively. B, freshly isolated iCD8^+^ DC and iCD8^−^ DC subsets were cultured. The culture supernatants were harvested at 72 h and tested for IL-10 and IL-12p70 by ELISA. Data are shown as mean±SD of each group. One representative experiment of three independent experiments with similar results is shown.*p<0.05, **p<0.01.

To further analyse the cytokine production by the DC subsets, we purified iCD8^+^ DC and iCD8^−^ DC using MACS columns. As shown in Figure 2A, the sorted DC subsets were in high purity. To assay the cytokines production pattern of the DC subsets, the purified iCD8^+^ DC and iCD8^−^ DC were cultured and the cytokine levels in the 72 h culture supernatants were measured by ELISA. As shown in Figure 2B, iCD8^+^ DC produced significantly higher level of IL-12p70 than iCD8^−^ DC, which produced more IL-10. Taken together, the results indicate that BCG immunization leads to a preferential expansion of CD8^+^ DC which shows a different profile of co-stimulatory surface molecules and cytokines from CD8^-^ DC.

### iCD8^+^DC Are More Efficient in Promoting Th1 (IFNγ) Cytokine Production While iCD8^−^ DC Are More Efficient in Promoting Th2 Cytokine (IL-4) Production by T Cells Primed with BCG *In Vivo*

**Figure 3:**
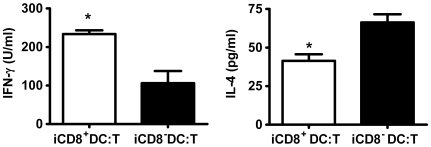
iCD8^+^ DC and iCD8^−^ DC subsets generate different patterns of BCG-driven T cell cytokine production. BCG-specific CD4^+^ T cells (5×10^5^) cell from BCG-infected mice were co-cultured with iCD8^+^ DC or iCD8^−^ DC (5×10^4^ cells/well) in the presence of HK-BCG. Culture supernatants were collected at 72 h. IFN-γ and IL-4 were measured by ELISA. The experiment was repeated twice showing similar results. *p<0.05.

Since the analyses shown above on co-stimulatory molecule expression and cytokines production suggest a functional difference of the DC subsets isolated from BCG immunized mice, We performed experiments to address this by co-culturing iCD8^+^ DC and iCD8^−^ DC subsets separately with CD4 T cells isolated from BCG primed mice. As shown in Fig. 3, iCD8^+^ DC promoted dominant Th1-cytokine (IFNγ) production, while the iCD8^−^DC induced a dominant Th2 (IL-4) cytokine response by antigen-specific CD4 T cells. The experiments using an in vitro approach demonstrated that the DC subsets were functionally different in directing BCG antigen-specific T cells.

### Adoptive Transfer of CD8^+^ DC, but Not CD8^−^ DC, Enhanced Bacterial Clearance and Reduced Pathological Reactions in the Infected Tissues Following Challenge Infection

**Figure 4:**
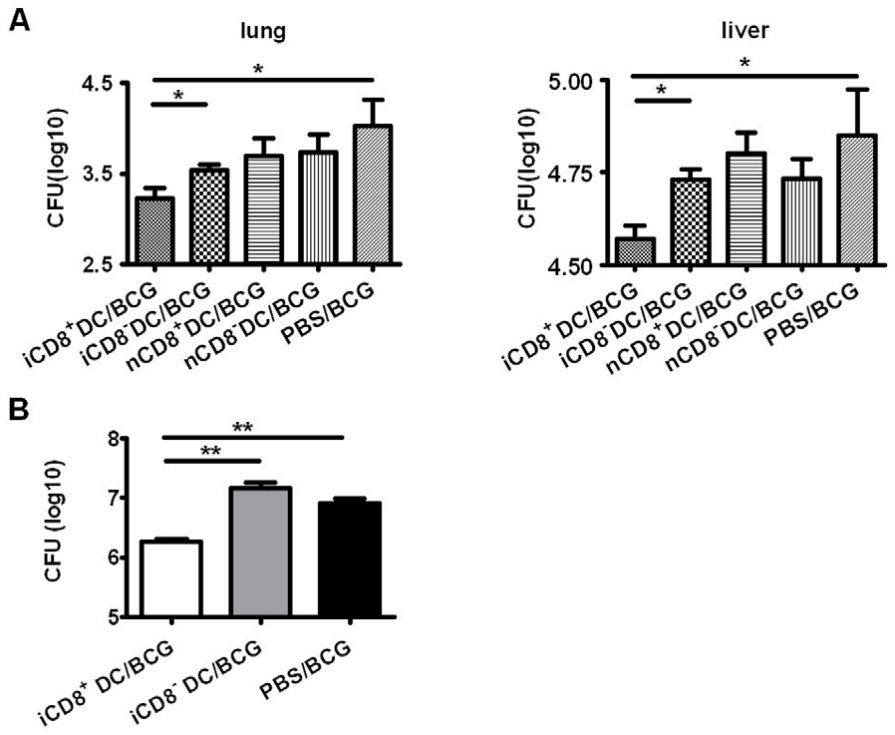
Adoptive transfer of iCD8^+^DC, but not iCD8^−^ DC, reduced bacterial growths in recipients following either i.v. or i.n. challenge infection. DC subsets were sorted from BCG-infected (i.v.) mice or naïve mice and adoptively transferred to syngeneic recipients (C57BL/6, n = 4/group) by i.v. (A&B, 5×10^5^ DC/mouse) or i.n. routes (C, 2×10^5^ DC/mouse). Mice were challenged with BCG through i.v. (A&B) or i.n. (C) routes, respectively, as described in Materials and Methods. All mice were sacrificed at day 21 post challenge infection. Homogenized lung or liver tissues were measured for BCG CFU. The CFUs of BCG were converted to logarithmic values and presented as mean±SD of each group. One representative experiment of three independent experiments is shown. * p<0.05;**, p<0.01.

To further examine the functional difference of the DC subsets, we adoptively transferred the iCD8^+^ DC and iCD8^−^ DC subsets and tested protection in the recipients of the different DC subsets to challenge infection. DC subsets were purified from BCG-infected mice at day 21 post immunization and were adoptively transferred i.v. to naïve C57BL/6 mice. At 7 days after cell transfer, the recipient mice were challenged i.v. with BCG. Mice that received PBS (sham treatment) or DC subsets from naive mice with the same challenge infection were used as controls. Twenty-one days after challenge infection, mice were sacrificed and the bacterial loads in the lung and liver were measured. As shown in Fig. 4, a significant reduction in tissue bacterial loads were observed in the mouse group receiving iCD8^+^ DC compared with the mice without DC transfer (sham treated control mice). In contrast, although the recipients of iCD8^−^ DC also appeared to have a trend of lower CFU in the lung and liver compared to PBS controls, the differences were not statistically significant. As controls, the recipients of either DC subsets from naïve mice (nCD8^+^ DC or nCD8^−^ DC) showed comparable levels of bacterial loads with the PBS treated control mice, therefore not generating protection.

**Figure 5:**
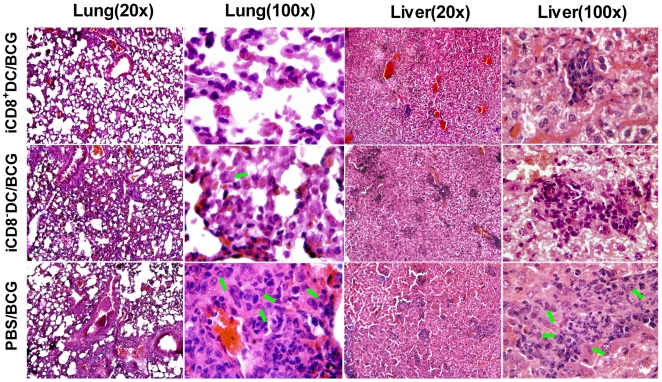
Significantly milder pathological changes in recipients of iCD8^ +^DC following intravenous challenge infection. Mice were treated/challenged as described in the legend to Figure 4 and analyzed for histopathological changes in lung and liver at day 21 post challenge infection by H&E staining. Low magnification(x20) and high magnification (x100) were shown respectively. Green arrows indicate typical epithelioid cells.

More remarkably, further histopathological analysis (Figure 5) showed that the recipient of iCD8^+^ DC only had mild pathological changes in the lung and liver. In contrast, the sham-treated mice and the recipients of iCD8^−^ DC showed much more severe pathological inflammatory reactions. Interestingly, although both sham-treated mice and iCD8^−^ DC recipients showed severe pathological reactions, their pattern of changes were different. Sham-treated mice showed multiple granulomas in both lung and liver tissues, characterized by dominant epithelioid cells (indicated by green arrows in Fig. 5) at centre with surrounding lymphocytes, neutrophil and monocytes in peribronchial and perivascular areas (lung) and peri-central vein areas of the hepatic lobules (liver). Multinucleate giant cells were also observed in the sham-treated mice. In contrast, the inflammation in iCD8^−^ DC recipients was diffused without notable granulomas and lack of typical epitheloid cells. The infiltrating cells in these mice were mainly neutrophils and mononuclear cell.

Since tuberculosis is mainly a pulmonary disease, we further analysed the protective function of the DC subsets using a model of intranasal challenge infection. DC subsets were adoptive transferred to recipient mice followed by i.n. challenge infection with live BCG. Similar to what observed in the i.v. infection model, the recipients of iCD8^+^ DC showed lest organism growth (Figure 4B) and mildest pathological changes (not shown) in the lung among the groups, exhibiting very few inflammatory cells following intranasal challenge. In contrast, evident pathological changes were found in the sham treated control group (multiple granuloma and heavy inflammation) and iCD8^−^ DC recipients (disseminated heavy inflammation). Taken together, the results indicate that only the CD8^+^ DC subset from infected mice can generate significant protective immunity in vivo. Moreover, since granuloma formation is a way for host to control infection (although not very efficient), the failure of iCD8^−^ DC recipients to have granuloma formation instead showing heavily diffused inflammation suggest it may promote an inflammatory reaction which is not protective.

**Figure 6:**
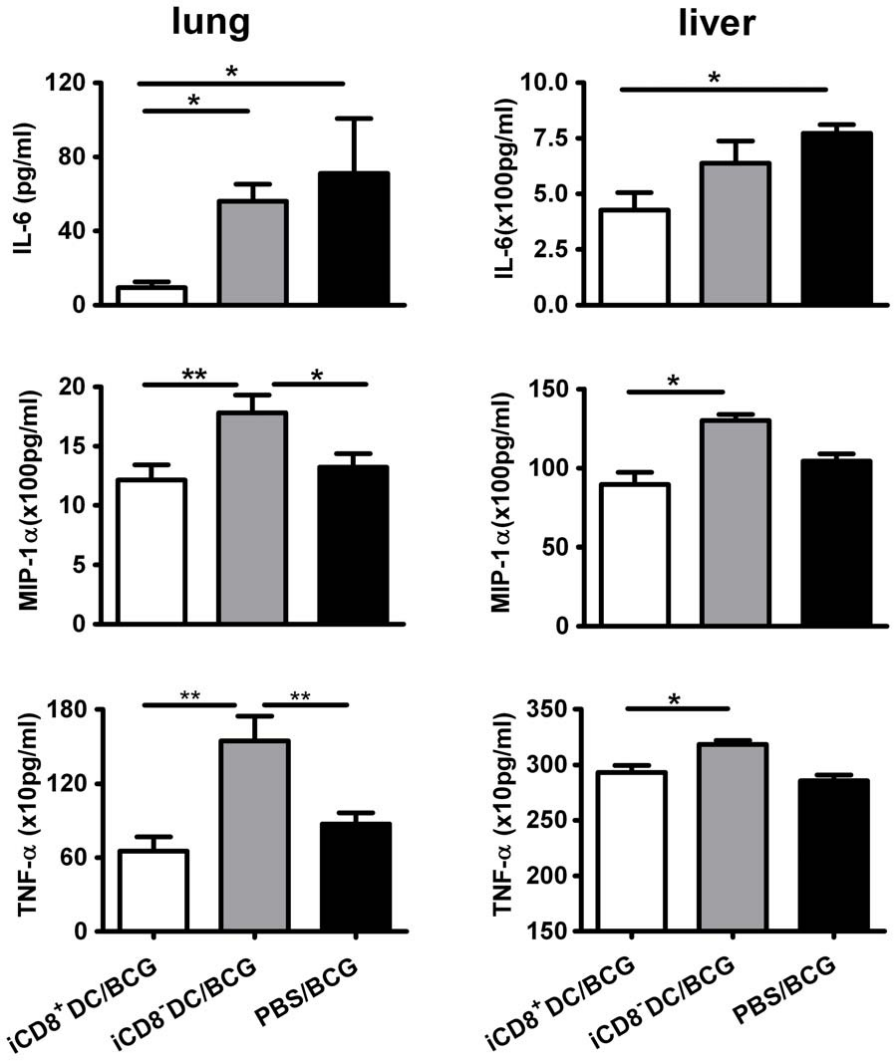
Pro-inflammatory cytokine/chemokine levels in local tissues in mice treated with different DC subsets following challenge infection. The mice were treated as described in the legend to Fig. 4 and the levels of IL-6, MIP-1α and TNF-α proteins in the homogenates of lung and liver tissues were measure by ELISA.

Since proinflammatory cytokine and chemokine responses contribute to the inflammation of local tissues and correlate with the degree of pathological reactions in some circumstances, we further measured the MIP-1α (CCL3), IL-6 and TNF-α concentration in the lungs and liver. In consistence with the lowest degree of inflammation in the iCD8^+^DC recipients (Figure 5), this group of mice showed the lowest levels of IL-6, MIP-1α and TNF-α response in the local tissues (Figure 6). In contrast, the recipient of iCD8^−^DC showed highest levels of these cytokines, even higher than the sham-treated, infected mice. Potentially, in the infected tissues many different types of inflammatory cells can produce these cytokines/chemokine including, but not limited to macrophages, DCs, and lymphocytes. The higher levels of these molecules in the lung of the iCD8^−^DC recipients suggest a non-protective or less protective severe inflammation in the lung, which is not efficient for clearing the infection but contributes to pathological changes. This data are consistent with the severe and diffused inflammation in the iCD8^−^DC recipients (Figure 5). The data suggest that the transfer of iCD8^+^DC, but not iCD8^−^DC, can reduce the pathological inflammatory responses in the local tissues.

### The Adoptive Transfer of iCD8^+^ DC, but Not iCD8^−^ DC, Enhances Type 1 Immune Responses Following Challenge Infection

**Figure 7:**
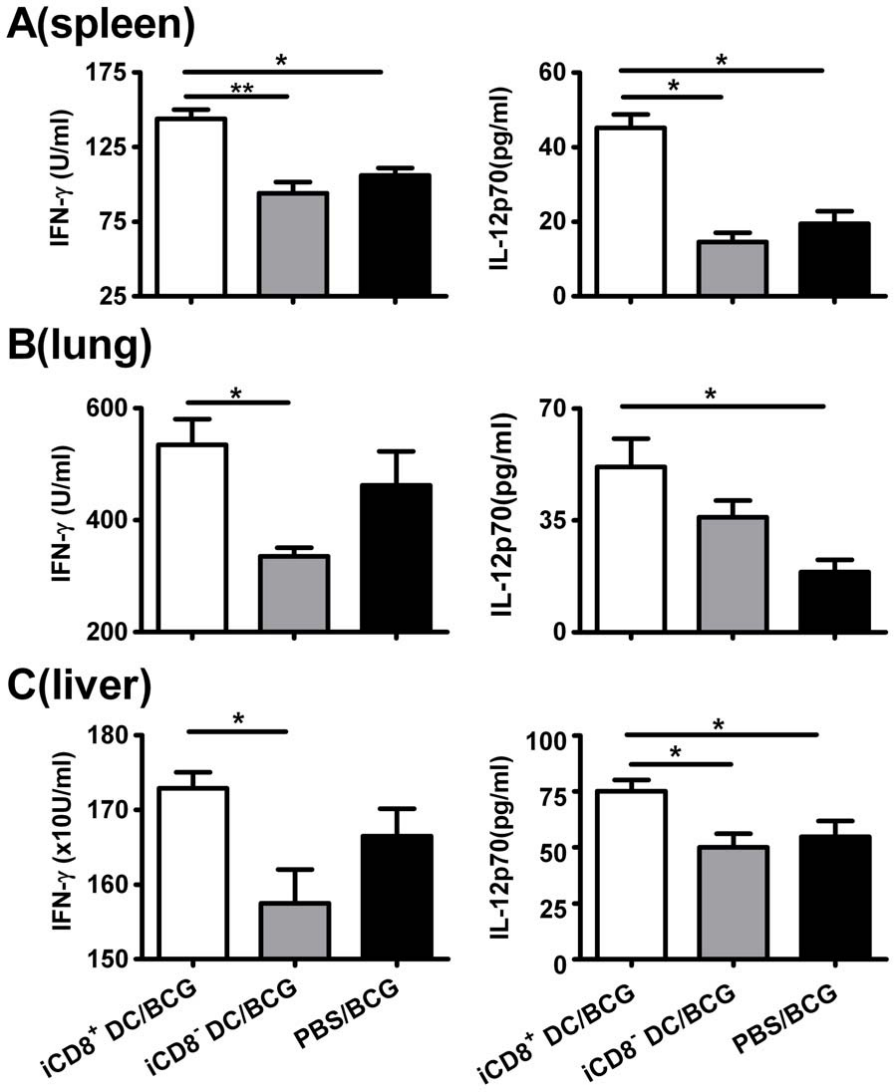
Effects of different DC subsets adoptive transfer on Th1-related cytokine production by recipient mice following challenge infection. **A:** The recipient mice (C57BL/6, n = 4/group) of i.v. adoptive transferred iCD8^+^ DC or iCD8^-^ DC subsets and PBS treated control mice were challenged i.v. with BCG and sacrificed at day 21 post challenge as described in Materials and Methods. A, splenocytes were cultured at the concentration of 7.5×10^6^ cells/well using HK-BCG as stimulator. Culture supernatants were harvested at 72 h and the cytokines were measured by ELISA. B&C, lungs and livers were homogenized in 10 ml cold protein-free D-PBS and centrifuged. The cytokines levels in the lung (B) and liver (C) were measured by ELISA. Data are presented as mean±SD of each group. One representative experiment of three independent experiments is shown. *p<0.05; **, p<0.01.

Since the type of immune responses plays a critical role in protection against mycobacterial infection, we further examined the T cell cytokine patterns and antibody responses in the recipients of different DC subsets in order to elucidate the mechanism underlying the difference in protection. As shown in Figure 7A, significant difference in the production of BCG driven type-1 related cytokines (IFN-γ and IL-12) by bulk cultured spleen cells was observed between the different groups following challenge infection. Specifically, the recipient of iCD8^+^ DC showed significantly higher IFN-γ production than the control mice without cell transfer. Analysis of cytokine patterns in the local tissues also showed higher IL-12 and IFN-γ levels in the lung (Figure 7B) and liver (Figure 7C) of the iCD8^+^ recipient mice compared to sham control groups and iCD8^−^ DC recipient mice. Serum antibody analysis showed low titers of BCG specific IgG2a and IgG1 antibodies in all the groups and no significant difference was observed among the groups (data not shown).

**Figure 8:**
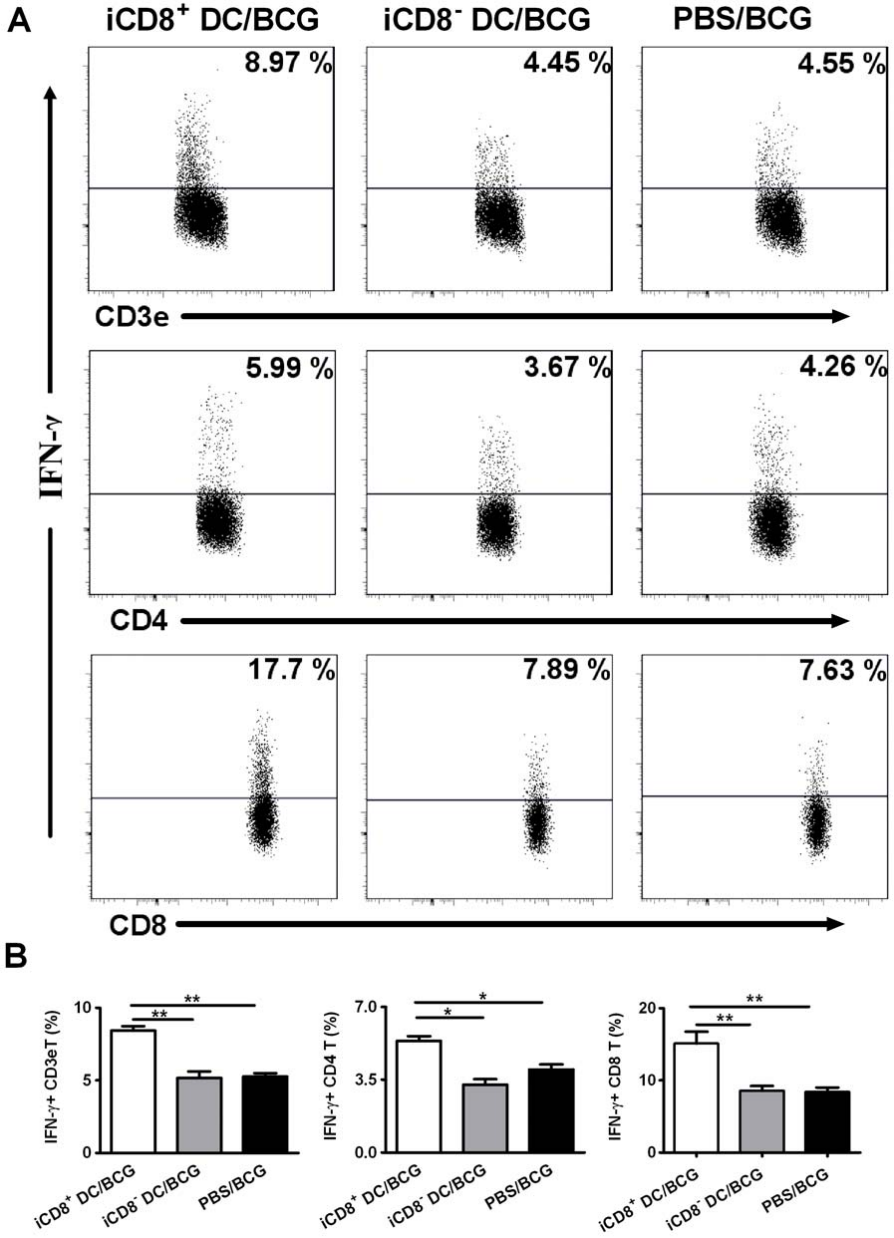
Intranasal adoptive transfer of iCD8^+^DC, but not iCD8^−^DC, enhanced IFN- γ production by T cell. Mice were adoptively transferred i.n. with iCD8^+^DC or iCD8^−^DC subsets followed by the challenge with BCG through i.n route as described in Materials and Methods. Draining lymph nodes was isolated aseptically and single cell suspension was prepared in cold staining buffer. Cells were co-stained with FITC-anti CD3ε, PE-anti CD4, PerCP-anti CD8 Abs and stained for intracellular IFN-γ using allophycocyanin -conjugated anti-IFNγ Ab as described in Materials and Methods. Cells were gated on CD3ε^+^T cells, CD3ε^+^CD4^+^T cell and CD3ε^+^CD8^+^T cell respectively and the percentage of positive cells for IFN-γ is indicated in upper right corner (A). Pooled data in each group are shown as mean±SD (B). One representative experiment of two independent experiments is shown. *p<0.05; **, p<0.01.

To further examine the local T cell responses, we performed intracellular cytokine staining of T cells from the draining lymph nodes following intranasal challenge infection. As shown in Figure 8, the adoptive transfer of iCD8^+^ DC induced more IFN-γ producing T cells than iCD8^−^ DC. In particular, recipients of iCD8^+^ DC mounted more than two fold higher IFN-γ producing CD8 T cells than the sham-treated mice. Similarly, more IFNγ–producing CD4 T cells were also found in the recipients of iCD8^+^ DC. In contrast, adoptive transfer of iCD8^−^ DC failed to enhance IFNγ response by either CD4 or CD8 T cell. Taken together, the results suggest that iCD8^+^ DC has strong ability to promote type-1 T cell responses to mycobacterial challenge infection, which may be the basis for its strong capacity to generate protection against both systemic and local challenge infections.

### DC Subsets Isolated from BCG Infected Mice Show Similar Levels of BCG Messages

**Figure 9:**
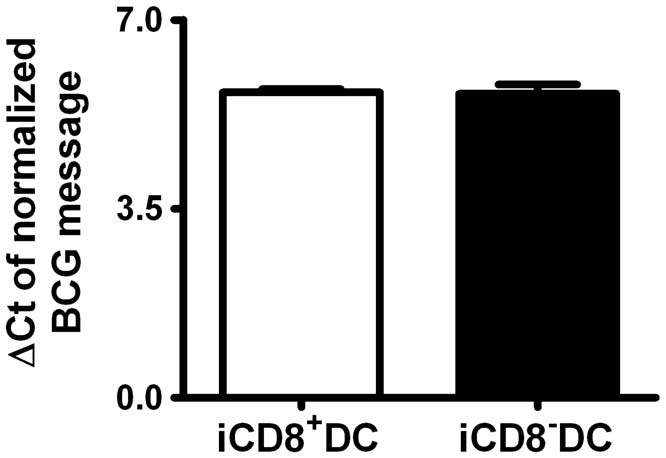
DC subsets from BCG-infected mice show similar BCG load. A real-time RT-PCR using the green fluorescent dye SYBR Green I was applied to determine the relative concentration of BCG mRNA in iCD8^+^DC and iCD8^−^DC. The BCG mRNA in different DC subsets was amplified as described in Materials and Methods. The BCG mRNA level was normalized to GAPDH mRNA in different DC subsets demonstrated as *ΔCt*. *ΔCt* = *Ct* (BCG)–*Ct* (GAPDH), the threshold cycle of a BCG and the threshold cycle of the corresponding GAPDH in the same sample.

A question which needed to be addressed was if the differences observed above for the DC subsets isolated from infected mice in generating protective immunity was due to the potential difference of the subsets in BCG loads, thus the amount of antigens carried. To answer this question, we further analyzed the bacterial loads of the CD8^+^DC and CD8^−^DC subsets from BCG infected mice using quantitative RT-PCR. As shown in Figure 9, the BCG mRNA levels in the two DC subsets demonstrated in real-time PCR analysis were comparable. The in vitro culture of either DC subsets showed negative results for viable BCG. The data suggest that the DC subsets are not significantly different in carrying BCG and possibly its antigens.

## Discussion

This report assessed the capacity of DC subsets in BCG infected mice in generating protective immunity against mycobacterial challenge infection. The data revealed that CD8^+^ DCs, but not CD8^−^DCs, from the infected mice are able to induce protection and reduce pathological reactions in the local tissues of the recipient mice. This conclusion is supported by the facts that the recipients of iCD8^+^ DCs exhibited significantly less bacterial growth, much milder histopathological changes and significantly stronger type-1 immune responses compared to the mice without cell transfer or received iCD8^−^DCs. To our knowledge, this is the first report showing the different capacity of DC subsets primed by in vivo immunization by BCG in reducing bacterial loads and pathological reactions following systemic and local mycobacterial challenge infection.

Several published studies have used DCs which are infected by BCG in vitro or are loaded with mycobacterial antigens to investigate the role of DC in inducing T cell immune responses and protection using cell adoptive transfer approaches [24], [25]. Demangel et al found that bone marrow-derived DCs that were infected with BCG showed increased expression/production of MHC class II antigens, co-stimulatory molecules and immune regulatory cytokines. Intratracheally adoptive transfer of these infected DCs induced a potent protection against aerosol Mtb challenge, similar to those induced by in vivo BCG immunization [24]. Gonzalez-Juarrero et al reported that lung DCs pulsed with Ag85 (LDC-Ag85) in vitro were able to prime naive CD4^+^ T cells in vivo after adoptively transfer [25]. However, the LDC-Ag85 recipients were not more resistant to Mtb challenge than those receiving DCs pulsed with an irrelevant protein, instead, showing more serious consolidation in the lung. Comparing to these reported studies, our study using DCs primed/infected with BCG in vivo addressed the function of DCs in a more physiological and relevant way. Most importantly, our data not only showed the critical role of DCs in BCG-induced protection against mycobacterial challenge infection, but the different capacity of DC subsets in this matter.

In theory, a potential possibility for the difference of the DC subsets in generating protective immunity is because the DC subsets are different in uptaking and/or hosting BCG thus leading to differential immune responses. This concern is reasonable because some early studies have shown a poor phagocytic capacity of CD8^+^DC in certain infections [26], [27]. However, our analysis on BCG gene expression in the two DC subsets showed similar levels of BCG mRNA in the isolated DC subsets (Figure 9). This is consistent with a reported study showing the similar efficiency of CD8^+^ DC and CD8^-^ DC in acquiring mycobacteria-derived antigens [28]. This is also consistent with other reports which demonstrate a similar efficacy of the CD8^+^ DC and CD8^−^ DC subsets in phagocytosis [29], [30]. Therefore, the difference in immune responses generated by the adoptive transfer of the DC subsets observed in our study is more likely reflecting a functional difference of the subsets in activating/modulating T cell responses rather than their difference in handling the microbes/antigens, especially considering the dramatic difference of the DC subsets in the expression of co-stimulatory molecules and the production of immunoregulatory cytokines.

The expression of co-stimulatory cell surface molecules and the production of cytokines have been shown to be the most important bases by which DCs modulate the function/polarization of T cell responses. Our data showing the significant differences of the DC subsets in the expression and production of these molecules and cytokines fit well with the functional difference of the DC subsets in inducing the different types of immune response and protection. In our study, a higher proportion of iCD8^+^ DC expressed costimulatory surface markers CD80 (65.66% vs. 17.43%), CD86 (57.43% vs. 30%) and CD40 (44% vs. 35%) compared with iCD8^−^ DC. So were the densities (MFI) of the expressed molecules. More importantly, iCD8^+^ DC predominantly produced Th1 promoting cytokine, IL-12p70 and IL-12p40 (not shown) while iCD8^−^ DC produced higher IL-10. This is inline with a reported study showing CD8^−^ DCs induced a Th2-type immune response, while the CD8^+^ DCs led to the Th1 differentiation [31]. IL-12 producing DC has been found to be powerful in generating protection against tuberculosis infection [32], [33]. IFN-γ is closely related to protective immunity to tuberculosis infection and is the principal macrophage-activating cytokine[34], which can stimulate the synthesis of reactive oxygen intermediates and nitric oxide by inducing production of phagocyte oxidase and nitric oxide synthase within lysosomes. IFN-γ induced production of reactive nitrogen intermediates is one of the most important mechanisms for controlling mycobacterial infections [35]. Therefore, the higher production of IL-12 by iCD8^+^DC and the subsequently enhanced IFN-γ production induced by the adoptive transfer of this DC subset is likely the major reason for the better protection of the iCD8^+^DC recipients observed in the present study. In addition to modulate CD4 T cell response, we also found a significantly higher IFN-γ production by CD8 T cell in the recipients of iCD8^+^DC. In fact, the difference in CD8 T cells for IFN-γ production by the different experimental groups was even more apparent than in CD4 T cells. This finding is inline with recent reports showing an exceptional ability of CD8^+^DC to present exogenous antigens through a process of cross-presentation [36], [37] and suggests the importance for enhancing CD8+ T cell responses in promoting host defense against mycobacterial infections.

A notable characteristic of iCD8^−^ DC observed in the present study is its dramatically higher production of IL-10 compared to that of iCD8^+^ DC. It has been reported that, although IL-10 knockout mice showed little difference in resistance to Mtb infection than wild-type mice[38], gene transgenic mice that overly expressed IL-10 exhibited increased reactivation of chronic infection[39]. Our data showed that adoptive transfer iCD8^−^ DC had no significant effect on BCG loads in the lung and liver following challenge infection. However, histological analysis showed that the inflammation in the lung and liver tissues of iCD8^−^ DC recipients was not only much heavier than the iCD8^+^ DC recipients but also more severe than the mice without previous DC transfer. Typical multinucleate giant cells were found around the granulomas in mice without DC transfer. However, the iCD8^−^ DC recipients showed more diffused inflammation. Granuloma formation is an important way for the host to control mycobacterial infection, although its efficiency is not absolute. The most successful control of infection would lead to resolution of inflammation without granuloma formation. However, the lack of granuloma formation with diffused inflammation observed in the iCD8^−^ DC recipients implies a lack of efficient control for the infection and the development of more severe pathological inflammation. The highest level of inflammatory cytokines/chemokine (IL-6, MIP-1α, TNF-α) in the iCD8^−^ DC recipients confirms the severe inflammation in these mice. Since granuloma in mycobacterial infection is a type-1 granuloma that is promoted by IFNγ and inhibited by IL-10, the higher IL-10 production by iCD8^−^ DC may be the basis for the significantly reduced granuloma formation in its recipients.

It should be noted, however, that the challenge infection used in the study is BCG instead of virulent *Mtb* strains which are the real cause of tuberculosis. It is possible that immunity against challenge by virulent Mtb requires additional parameters. Therefore cautions are needed in interpreting the data from this study. Further study using virulent *Mtb* as challenge infection would be important. On the other hand, since many similarities in immune responses to BCG and *Mtb* have been reported and BCG has been used for modeling host defense against *Mtb* in many studies, the data from current study are very useful for guiding future studies and for understanding host defense mechanisms against *Mtb* infection.

In conclusion, the present study has demonstrated that different DC subsets from BCG-infected mice are different in capacity to generate protective immunity. The functional difference may be related to their expression of co-stimulatory molecules and cytokine production patterns. The data suggest that proper targeting and/or modulating “right” DC subset may have the potential to improve the efficiency of vaccination to generate protective immunity. This knowledge may have implications in the development and improvement of the approaches for prevention and therapy of tuberculosis infection and subsequent sequelae.
